# Leishmania spp. in a cutaneous histiocytoma of an old dog

**DOI:** 10.1002/dc.24839

**Published:** 2021-07-29

**Authors:** Jari Zambarbieri, Claudio Pigoli, Mario Caniatti, Paola Scarpa

**Affiliations:** ^1^ Department of Veterinary Medicine University of Milan Lodi Italy; ^2^ Laboratorio di Istologia Istituto Zooprofilattico Sperimentale della Lombardia e dell'Emilia Romagna (IZSLER) Brescia Italy

## 1 INTRODUCTION

The description of the unique cytological features of a skin lesion in an old dog affected with leishmaniasis is here reported.

In a nodular lesion on a metacarpus the coexistence of histiocytic cells suggestive of histiocytoma and *Leishmania* spp. amastigotes, both on the background and in the neoplastic cells, were found.

## 2 CASE REPORT

A 10‐year‐old male Italian Volpino crossbreed dog was referred for a second opinion for not‐responsive anemia. The dog had a history of travel across Europe, including Spain. A month before, the dog was visited in the Canary Islands for lethargy, dysorexia and vomit lasting for about 2 months with a worsening in the last week. The Complete Blood Count (CBC) revealed severe normocytic normochromic anemia [RBC 1.58 × 10^6^/μl (RI 5.5–8.5), Hgb 3.4 g/dl (RI 12–18), Hct 10.36% (RI 37–55), MCV 66 μ^3^ (60–77), MCH 21.8 pg (19.5–24.5) MCHC 33.1 g/dl (31–38) RDW 20.1% (12.5–16)] and thrombocytopenia (44,000/μl, RI 150–500); biochemistry profile identified a significant hyperproteinemia (9.4 mg/dl, RI 5.4–8.2) due to hyperglobulinemia (6.3 mg/dl, RI 2.3–5.2). The owner referred that the day after the dog undergo blood transfusion, and started antibiotic and corticosteroid therapy. A week later hematological parameters were quite improved, but anemia was still present [(RBC 3.47 × 10^6^/μl (RI 5.5–8.5), Hgb 7.9 g/dLl (RI 12–18), Hct 21.75% (RI 37–55), MCV 63 μ^3^ (60–77), MCH 22.7 pg (19.5–24.5) MCHC 36.3 g/dl (31–38) RDW 18.3% (12.5–16)]. No other medical reports were available.

**FIGURE 1:**
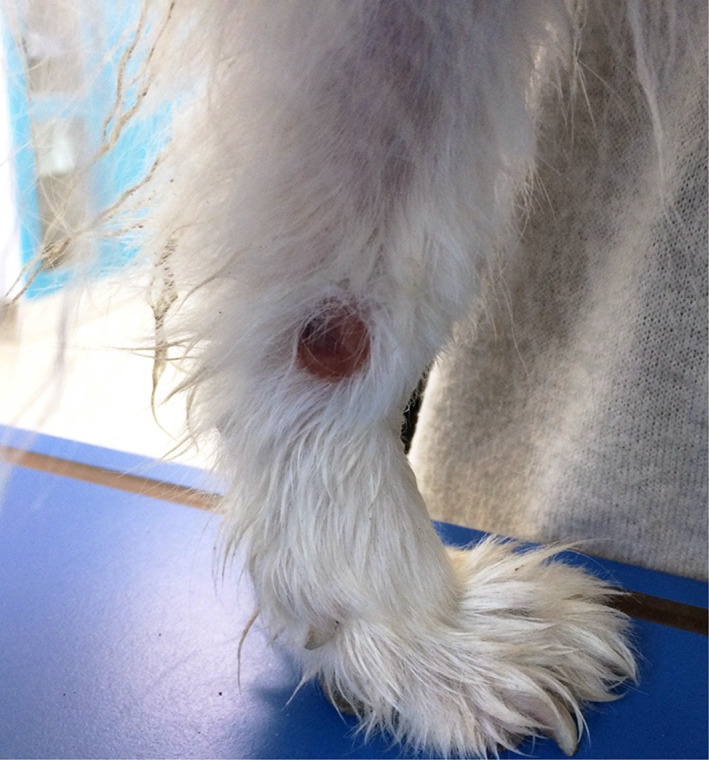
Macroscopic appearance of the lesion: a roundish (2.5 × 2.5 cm), raised, pinkish, soft, and alopecic mass on the lateral surface of the right metacarpus is evident

When the dog was referred to our Hospital, clinical examination revealed poor body condition (BCS 3/9), pale mucous membranes and a cutaneous alopecic mass on the lateral surface of the right metacarpus. The mass was roundish (2.5 × 2.5 cm), raised, pinkish, soft and alopecic (Figure 1). Fine‐needle aspiration of the mass was performed for cytologic evaluation.

**FIGURE 2:**
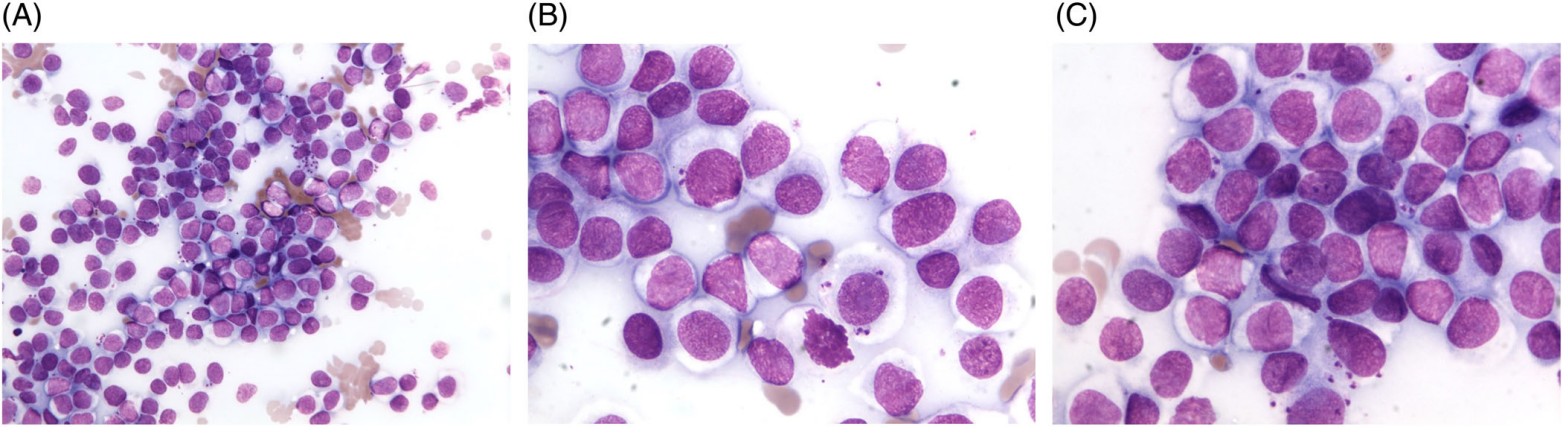
Microscopic appearance of the lesion (May‐Grünwald Giemsa, A: 40×, B,C: 100×): the specimen was mainly composed by atypical round cells with distinct cell borders, a moderate amount of homogeneous light‐blue cytoplasm, and round to oval, central to eccentric nuclei with finely stippled chromatin and inconsistent nucleoli; few mitoses were present. In the cytoplasm of some neoplastic cells a variable number of rounds to oval, 2.5–5 × 1.5–2 μm, *Leishmania* spp. amastigotes are present

The cytological specimen was highly cellular and moderately hemodiluted. It was composed of a neoplastic population of round cells admixed with occasional small mature lymphocytes. Neoplastic cells were roundish, 18–22 μm in diameter, with distinct cell borders and variable nuclear‐cytoplasmic ratio. Cells had moderate to abundant homogeneous light‐blue cytoplasm. Nuclei were round to oval, occasionally indented, 12–16 μm in diameter, central to eccentric, with finely stippled chromatin and inconsistent nucleoli. Anisocytosis and anisokaryosis were moderate. Few mitoses were seen. In addition, the cytoplasm of some neoplastic cells contained a variable number of rounds to oval, 2.5–5 × 1.5–2 μm, *Leishmania* spp. amastigotes characterized by a small round nucleus and a rod‐shaped blue kinetoplast; *Leishmania* spp. amastigotes were also seen free in the background. Based on cytological findings, a diagnosis of canine cutaneous histiocytoma and leishmaniosis was made (Figure 2).

Additionally, a new CBC confirmed a persistent severe slightly regenerative anemia [RBC 2.45 × 10^6^/μl (RI 5.7–8.8), Hgb 6 g/dl (RI 12.9–18.4), Hct 18% (RI 37.1–57), MCV 73.5 μ^3^ (60–77), MCH 24.5 pg (19.5–24.5) MCHC 33.3 g/dl (31–36) RDW 22.7% (11.9–18.5), reticulocytes 84,000/μl (RI <60), corrected reticulocyte percentage 1.4% (RI >1 in case of anemia)]. Urinalysis revealed low specific gravity (1018, RI 1020–1060) and significant proteinuria (UPC 0.66, RI <0.5). A *Leishmania infantum* IFAT (IgG) titer was positive at a dilution <1:1280 (RI < 1:80) while IgM titer was negative.

Due to cytological identification of amastigotes and the high titer of anti‐*Leishmania* spp. antibodies, the dog was considered sick (stage C) according to a proposed clinical classification scheme and underwent treatment with meglumine antimoniate.^1^

The skin lesion underwent to size reduction within few weeks, before a new move of the dog owner.

## 3 DISCUSSION

Leishmaniasis is a common canine vector‐borne disease endemic in central and western Mediterranean basin whose causative agent is *Leishmania infantum* (*Leishmania donovani* complex).^2^ The disease is characterized by a wide spectrum of clinical presentations and clinicopathologic abnormalities. Clinical signs include poor nutritional state, muscular hypotrophy, lymph nodes enlargement, pale mucous membranes, skin lesions, primarily desquamative dermatitis and less frequently ulcerative, popular or nodular dermatitis, onychopathy, ocular and palpebral alterations and lameness secondary to polyarthritis.^1^, ^2^ The main laboratory abnormalities include normocytic normochromic anemia, thrombocytopenia, hyperproteinemia with polyclonal gammopathy, and proteinuria; all of them were present in our case, even if a serum protein electrophoresis was not available. Briefly, anemia is multifactorial, including in the pathogenesis the presence of a chronic disease, a possible hemolytic component and a possible renal failure; thrombocytopenia is mainly due to an immune‐mediated peripheral destruction; total proteins and globulin are increased due to the severe systemic inflammatory response; proteinuria is secondary to the deposition of immune complexes in the glomerulus.^1^, ^3^

Canine cutaneous histiocytoma is a common benign neoplasm occurring as a solitary, alopecic, dome‐shaped lesions often undergoing spontaneous regression. Although histiocytomas occur in dogs of all ages, they are usually found in patients <3‐years‐old. In our case, macroscopic and cytologic features were consistent with histiocytoma, although the age was not typical.^4^ Other differential diagnosis included inflammatory, such as abscess or granuloma, or neoplastic condition, such as mast cells tumor or epithelial neoplasia; these have been excluded due to the absence of other cell populations different from histiocytic cells.

Nodular lesions are uncommon in canine leishmaniasis and are usually characterized by pyogranulomatous inflammation associated with moderate to severe lymphoplasmacytic infiltration. In our case, *Leishmania* amastigotes were found in the background and also in the cytoplasm of some neoplastic histiocytic cells and, to our knowledge, this is the first report of this coexistence. Interestingly, in the literature only a case report describing the presence of *Leishmania* amastigotes in a histiocytic tumor, in particular a human fibrous histiocytoma, is reported.^5^

The coexistence of *Leishmani*a and neoplasia has been reported in humans and animals: in dog *Leishmania* and neoplasia have been documented in a variety of neoplastic conditions including lymphoma, cutaneous transmissible venereal tumor, splenic hemangiosarcoma, soft tissue sarcoma, and adrenocortical adenoma. In some tumors, such as transmissible venereal tumor, sarcoma, small‐cell lymphoma, adrenocortical adenoma and perianal adenoma, amastigotes were identified not only in the macrophagic cells but also within the neoplastic cells, as occurred in the case presented.^6^, ^7^, ^8^, ^9^, ^10^

The relationship between parasites presence and neoplasia is not yet clearly understood, although it is evident that leishmaniasis can directly or indirectly affect the presentation, diagnosis and course of malignant disorders. Among the proposed theories, the chronic inflammation induced by the parasites, the leishmaniosis interference with the local systemic immune system, and a direct involvement of leishmaniosis in the pathogenesis of cancer are those considered most relevant.^11^

Similarly to that reported, in our case, the pathogenesis of the coexistence between neoplastic cells and *Leishmania* spp. could be due to two of these proposed mechanisms. In the first scenario, the coexistence was consequent to the inoculation of amastigotes by the phlebotomine vectors in a pre‐existent neoplastic lesion confirming the nonspecific tropism of the parasite.^5^ In the reported case, the cutaneous lesion has not been identified by the owners before the onset of clinical signs. However, the rapid growth of histiocytic tumors does not allow to exclude the pre‐existence of the neoplastic lesion certainly.

The second possible explanation is that the interference of the inoculated pathogen with the local immunity ultimately led to histiocytic tumor development.^11^

This finding confirms the well‐reported ability of *Leishmania* spp. to coexist within neoplastic cells, although the exact pathogenesis of such finding is not yet completely known.

## 4 CONCLUSION

*Leishmania* spp. amastigotes have been identified in several benign and malignant neoplastic lesions in humans and dogs; among these, canine histiocytoma has now to be considered.

## CONFLICT OF INTEREST

The authors declare no potential conflict of interest.
